# Prediction Model for 30-Day Mortality after Non-Cardiac Surgery Using Machine-Learning Techniques Based on Preoperative Evaluation of Electronic Medical Records

**DOI:** 10.3390/jcm11216487

**Published:** 2022-11-01

**Authors:** Byungjin Choi, Ah Ran Oh, Seung-Hwa Lee, Dong Yun Lee, Jong-Hwan Lee, Kwangmo Yang, Ha Yeon Kim, Rae Woong Park, Jungchan Park

**Affiliations:** 1 Department of Biomedical Informatics, Ajou University School of Medicine, Suwon 16499, Korea; 2 Department of Anesthesiology and Pain Medicine, Samsung Medical Center, Sungkyunkwan University School of Medicine, Seoul 03181, Korea; 3 Department of Anesthesiology and Pain Medicine, Kangwon National University Hospital, Chuncheon 24289, Korea; 4 Rehabilitation & Prevention Center, Heart Vascular Stroke Institute, Samsung Medical Center, Sungkyunkwan University School of Medicine, Seoul 03181, Korea; 5 Department of Biomedical Engineering, Seoul National University College of Medicine, Seoul 03080, Korea; 6 Center for Health Promotion, Samsung Medical Center, Sungkyunkwan University School of Medicine, Seoul 03181, Korea; 7 Department of Anesthesiology and Pain Medicine, Ajou University School of Medicine, Suwon 16499, Korea

**Keywords:** risk, machine learning, mortality, surgery, artificial intelligence, prognosis

## Abstract

Background: Machine-learning techniques are useful for creating prediction models in clinical practice. This study aimed to construct a prediction model of postoperative 30-day mortality based on an automatically extracted electronic preoperative evaluation sheet. Methods: We used data from 276,341 consecutive adult patients who underwent non-cardiac surgery between January 2011 and December 2020 at a tertiary center for model development and internal validation, and another dataset from 63,384 patients between January 2011 and October 2021 at another center for external validation. Postoperative 30-day mortality was 0.16%. We developed an extreme gradient boosting (XGB) prediction model using only variables from preoperative evaluation sheets. Results: The model yielded an area under the curve of 0.960 and an area under the precision and recall curve of 0.216, which were 0.932 and 0.122, respectively, in the external validation set. The optimal threshold calculated by Youden’s J statistic had a sensitivity of 0.885 and specificity of 0.914. In an additional analysis with balanced distribution, the model showed a similar predictive value. Conclusion: We presented a machine-learning prediction model for 30-day mortality after non-cardiac surgery using preoperative variables automatically extracted from electronic medical records and validated the model in a multi-center setting. Our model may help clinicians predict postoperative outcomes.

## 1. Introduction

Surgical procedures are required for various health conditions. Worldwide, more than 300 million major surgeries are performed yearly [1]. Advances in surgical and anesthetic techniques have dramatically improved perioperative care and widened candidates for surgical procedures, particularly for older patients with more risk factors [2,3]. Postoperative mortality is now accepted as one of the leading causes of death in developed countries, and the balance between the risk and benefit of surgery is of clinical significance [4]. Thus, perioperative risk management has long been actively investigated [5]. The adoption of an electronic medical record system has enabled clinicians to efficiently organize relevant patient factors into a single document. Still, the adequate interpretation of these comprehensive data is a challenge. Furthermore, surgical patients often have coexisting risk factors that confound risk assessment.

Machine-learning techniques recently gained attention for handling numerous variables in nonlinear and interactive ways [6]. They have been actively used to evaluate predictors in various fields including medicine [6,7]. Previous studies reported that artificial intelligence has excellent performance for predicting postoperative mortality, and these models included intraoperative variables and were designed for real-time prediction during surgical procedures [8,9]. However, it is often unenviable for clinicians to decide on surgery based on preoperative risk and benefit. Thus, this study used a preoperative evaluation sheet to develop a prediction model based on machine-learning techniques. A preoperative evaluation sheet is a final document from which an attending anesthesiologist can gather a patient’s entire medical record related to surgical outcomes, allowing them to determine an anesthetic plan. The format of preoperative evaluation documents may vary between centers, but they commonly include well-known risk factors such as age, type of surgical procedure, results of blood laboratory tests, and underlying disease. In this study, we developed a prediction model for 30-day mortality after non-cardiac surgery from a single center and validated it with data from another center. Our results may offer the possibility of implementing a prediction model into an electronic medical record system in which clinicians can monitor mortality risk in real-time during preoperative evaluation and detect modifiable factors conveniently.

## 2. Methods

### 2.1. Study Population

This study used datasets from two different centers. For model development and internal validation, we used data from 276,341 consecutive adult patients who underwent non-cardiac surgery between January 2011 and December 2020 at our institution in Seoul, South Korea. Each patient’s preoperative evaluation sheet was automatically retracted using the Clinical Data Warehouse Darwin-C, which enables investigators to retrieve de-identified medical information. We collected data from 63,384 adult patients who underwent non-cardiac surgery between January 2011 and October 2021 at another center for the external validation set. Baseline characteristics were compared according to 30-day mortality, and details of the retained variables were also described.

Data use was approved by the Institutional Review Board at our institution (SMC 2022-06-132) and the Institutional Review Board of another center (AJIRB-MED-MDB-21-662). Informed consent from individual patients was waived because both registries were curated in a de-identified form. This study was conducted following the Declaration of Helsinki, and the results were reported according to the Strengthening the Reporting of Observational Studies in Epidemiology reporting guideline. The datasets generated during and/or analyzed during the current study are not publicly available but are available from the corresponding author at reasonable request.

### 2.2. Model Development

Data were divided into development, tuning, and internal validation datasets (70%:10%:20%) using a random split based on patients. No patient was assigned to both datasets. The development dataset was used for model building, and a hyperparameter was selected based on the tuning dataset. The internal validation dataset was only used for validating model performance, not used for model development and hyperparameter selection. Data from another center were employed as an external validation dataset.

We used only variables from the preoperative evaluation sheet for our models. In both hospitals, medical records and assessments related to anesthesia or surgery were organized by an anesthesiologist into a single preoperative evaluation document. Inspecting the data from the preoperative evaluation sheet, investigators at each hospital retrieved variables, including demographic data, underlying diseases, blood laboratory tests, physical examination, and the American Society of Anesthesiologists (ASA) physical status [10]. After extracting variables, we normalized continuous variables in each dataset using standard normalization. Missing data were imputed with the missforest algorithm [11].

The model endpoint was postoperative mortality within 30 days. We joined information from the National Population Registry of the Korea National Statistical Office to identify mortalities outside the hospital.

We developed a prediction model by utilizing extreme gradient boosting (XGB) [12], random forest (RF) [13], lasso logistic regression (LR) [14], and naïve Bayes (NB) [15] algorithms. We adjusted the hyperparameters based on Bayesian optimization during model building. Additionally, the final model algorithm was selected based on the area under the receiver operating characteristic curve in the tuning dataset of each algorithm-based model.

For comparison, we also developed a baseline model that mimics the classical clinical scoring system. The baseline model is a logistic regression model using only limited variables such as age, sex, body mass index, underlying disease, and ASA class.

### 2.3. Model Performance

For model evaluation, we calculated the area under the precision and recall curve (AUPRC) and the area under the receiver operating characteristic curve (AUROC) from the total population of internal validation and external validation datasets. We also calculated sensitivity, specificity, PPV (positive predictive value), and NPV (negative predictive value) with a cut-off point from Youden’s J statistic in the development dataset.

To validate calibration between the model’s predicted score and the proportion of clinical outcomes, we illustrated a calibration plot from the internal and external validation dataset and calculated the calibration metrics of the Brier score and integrated calibration index (ICI) [16,17].

To demonstrate the robustness of the machine-learning model, we conducted several sensitivity analyses. We divided patients into subgroups based on sex and age. Then, we observed model performance for each group. The subgroup criteria were male and female (sex) and <30, 30–40, 40–50, 50–60, and >60 years (age).

Additionally, model performance is sensitive to dataset distribution. To correct the imbalance of outcomes, we randomly down-sampled patients without 30-day mortality from the external validation dataset while preserving all patients with 30-day mortality. Finally, we created a dataset with ratios of balanced patients without outcomes versus patients with outcomes (1:1, 1:2, 1:4, 1:9) and calculated the AUROC value from the dataset. We also bootstrapped by down-sampling 1000 times to obtain the mean AUROC value and the confidence interval.

### 2.4. Model Interpretation

We utilized the SHapley Additive exPlanations (SHAP) summary graphic to interpret the model [18]. The SHAP value represents the effect of each characteristic on postoperative mortality by calculating a weighted average and marginal distribution. All variables are fixed except for one feature. Two SHAP-based plots were generated: the SHAP bar plot and the SHAP beeswarm plot. Both plots sort features based on their importance. The SHAP bar plot shows the importance of features in the prediction model by calculating the mean absolute SHAP value of features. The SHAP beeswarm plot represents each patient by a single dot on each variable line. The horizontal position denotes the strength of the correlation between the feature and the result. Variable specific SHAP values >0 indicate an increased risk of negative outcomes. The right side of the plot indicates where the SHAP value is >0.

### 2.5. Statistical Analyses

Baseline characteristics of patients with and without postoperative mortality were compared. We evaluated continuous variables with Student’s *t*-test and categorical features with the chi-square test. The 95% confidence interval (CI) for AUROC was calculated by the DeLong test. Analyses were performed using R 4.1.0 (R Core Team, Vienna, Austria).

## 3. Results

### 3.1. Baseline Characteristics

**Figure 1:**
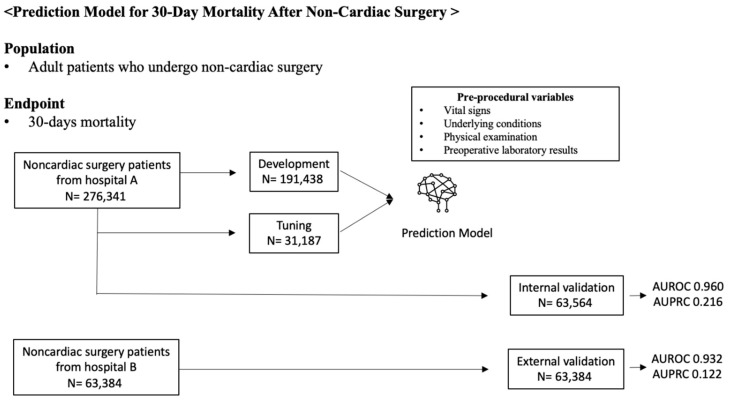
Overview of study. AUROC denotes the area under the receiver operating characteristic curve). AUPRC indicates the area under the precision and recall curve.

A total of 276,341 patients were included in the dataset model development. Among these, postoperative 30-day mortality was reported in 441 (0.16%) patients. We described the overview of the study in Figure 1, and all variables are described in Table 1.

Table 2 shows the baseline characteristics of patients with and without 30-day mortality in the development dataset. Patients with 30-day mortality were predominantly male, older, had higher ASA physical status classification, and tended to have lower blood pressure, temperature, and higher pulse rate. The external validation set included 63,384 patients. Baseline characteristics of the external dataset are summarized in Supplementary Table S1.

### 3.2. Model Performance

**Figure 2:**
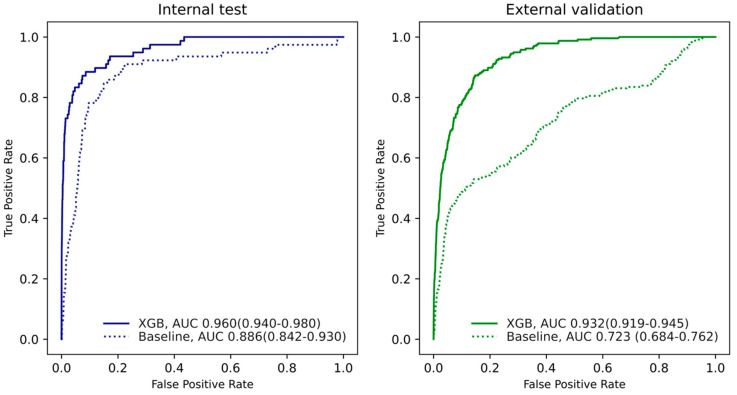
AUROC (area under the receiver operating characteristic curve) and AUPRC (area under the precision and recall curve) plots in the internal validation test set (Samsung medical center) and external validation dataset (Ajou university medical center). XGB (extreme gradient boosting algorithm) denotes the main model of our study based on various variables from preoperative evaluation sheets. Baseline denotes logistic regression model with only sex, age, weight, height, American Society of Anesthesiologists (ASA) class, and underlying disease.

The AUROCs for the XGB, RF, LR, and NB algorithms are described in Supplementary Table S2. Because the AUROC for the XGB model is higher than the other algorithms in the tuning dataset, we selected the XGB algorithm for the final model. For the internal validation set, the XGB-based mortality prediction model yielded AUROC = 0.960 (95% CI: 0.940–0.980) and AUPRC = 0.216. Model performance for the external dataset was AUROC = 0.932 (95% CI: 0.919–0.945) and AUPRC = 0.122 (Figure 2). Otherwise, the baseline model yielded AUROC = 0.886 (95% CI: 0.842–0.930) and AUPRC = 0.014. Model performance for the external dataset was AUROC = 0.723 (95% CI: 0.684–0.762) and AUPRC = 0.021. The XGB model showed a superior performance difference of more than AUC 0.2 in external validation. Additionally, the XGB model’s AUPRC was 0.1 or more in internal validation and 0.2 or more in external validation

**Figure 3:**
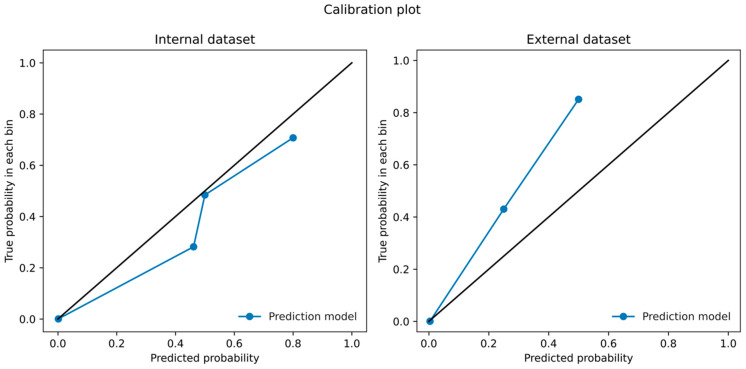
Calibration plot between model-predicted probability and true probability.

With an optimal threshold calculated by Youden’s J statistic, the model yielded sensitivity = 0.885 and specificity = 0.914, PPV 0.082, NPV = 0.999 for internal validation and sensitivity = 0.873, specificity = 0.853, PPV = 0.167, NPV = 0.997 for external validation. In calibration analysis, the Brier score and ICI were 0.0015 and 0.0044 for internal validation and 0.0036 and 0.0017 for external validation. The difference between the model’s predicted score and the proportion of clinical outcomes is illustrated in Figure 3.

### 3.3. Subanalyses

Balanced datasets created from under-sampling yielded a mean AUROC ≥ 0.932. The model performance of all down-sampled datasets is described in Supplementary Figure S1.

The model yielded an AUC ≥ 0.910 for all age groups and genders in subgroup analyses. Patients >60 years had the worst model performance across all sexes and compared with all other age groups. Detailed model performance for all subgroups is described in Table 3.

### 3.4. Model Interpretation

**Figure 4:**
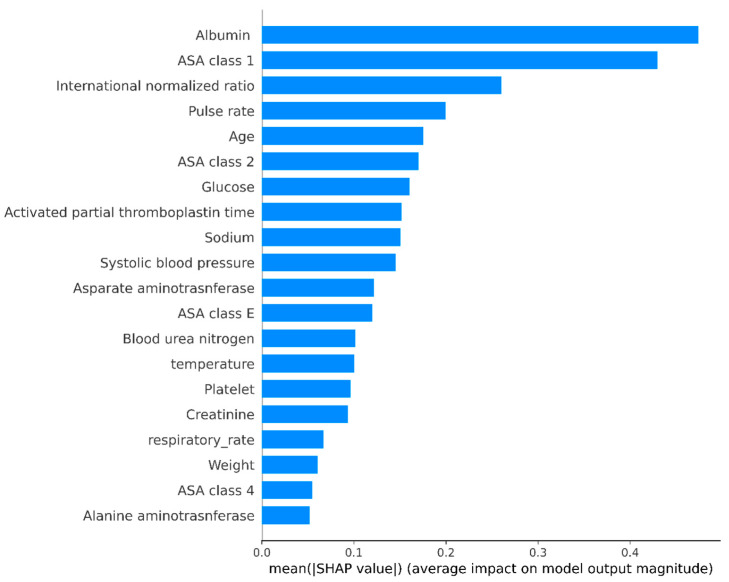
SHapley Additive exPlanations (SHAP) bap plot shows the importance of features in the prediction model by calculating the mean absolute SHAP value for each feature.

**Figure 5:**
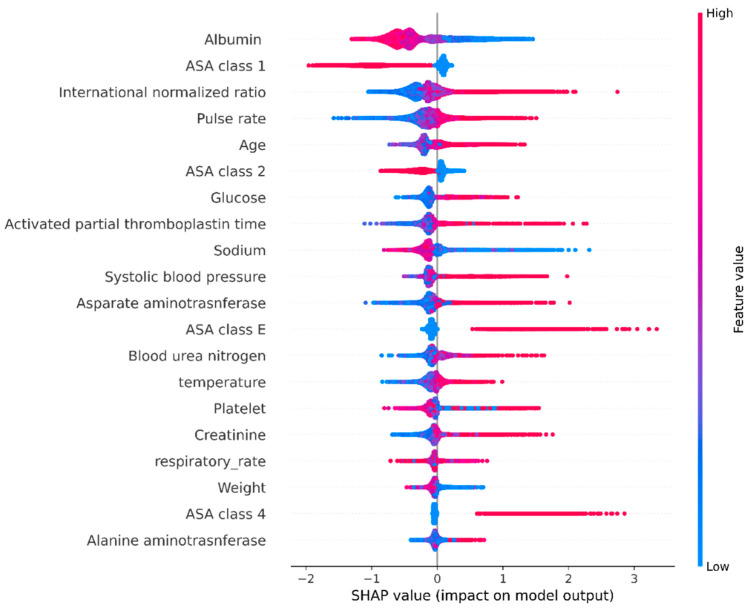
SHapley Additive exPlanations (SHAP) beeswarm plot shows a summary of how the top features in a dataset impact the model’s output. A red color means a high feature value. The blue color means a low feature value. Each point represents an individual person. The horizontal position of each point shows the impact of the feature on the model’s prediction. For example, in the case of ASA (American Society of Anesthesiologists) class 1, a high feature value (the red color) influences the model to predict less death. Conversely, in the case of age, the high feature value affects the model to predict more death.

Based on the prediction model results, we generated a SHAP bar plot (Figure 4) and a SHAP beeswarm plot (Figure 5). In the SHAP bar plot, the top five variables were albumin (0.47), ASA physical status 1 (0.43), international normalized ratio (0.26), pulse rate (0.2), and age (0.18). The SHAP beeswarm plot shows that lower serum albumin, sodium, and body weight were associated with higher mortality. Higher levels of glucose, aPTT, BUN, and creatinine were also associated with increased mortality.

## 4. Discussion

This study demonstrated a prediction model for 30-day mortality after surgery based on preoperative evaluation sheet data that are automatically retracted from electronic medical records. The preoperative evaluation sheet includes common risk factors for surgery, and our model achieved an excellent predictive value with AUROC > 0.9. Our findings suggest that implementing a prediction model using electronic medical records could provide a real-time estimation of the risk for postoperative mortality. This can be clinically helpful because some of the factors associated with increased risk, such as results of blood laboratory tests, may be modifiable.

Several scoring systems for postoperative outcomes have been suggested previously. The first was the preoperative ASA physical classification system developed in 1963 [10], followed by the Surgical Apgar score, which consists of three simple intraoperative factors and offers adequate performance [19]. However, combining pre and postoperative factors did not substantially improve prediction [20]. There have been further attempts to develop a more objective and accurate scoring system by adopting new statistical methods, such as logistic regression analysis [21]. The incorporation of machine-learning techniques enabled the interpretation of pre and intraoperative factors and improved model performance [8,9]. Our results demonstrate that a machine-learning prediction model can achieve a proper predictive value based on factors automatically extracted from preoperative documentation. The strength of our machine-learning technique-based prediction model is a comprehensive interpretation of preoperative factors, which resulted in a better predictive performance than a simple regression model. In addition, pre-existing models tended to be focused on particular groups of patients such as cardiac surgery, chronic patients, cancer patients, or critically-ill patients, but our model may be more conveniently used by automatically estimating risk based on electronic medical records regardless of surgery types.

This study focused on predicting mortality 30 days after surgery. Surgical procedures have a wide variety of aims. However, it is not easy to justify the high risk of 30-day mortality according to potential benefits or the aims of specific surgical procedures unless it is a palliative procedure during terminal state. Our prediction model did not retain palliative surgery because our institution is focused on curative care and patients are usually transferred for palliative care. Our original dataset was from one of the nationwide largest centers and retained nearly all types of curative surgeries for cancer and degenerative disorders. Furthermore, the model was validated in a large trauma center. Therefore, our model could be applied in nearly all types of surgery that need risk stratification. Curative surgical procedures are now actively considered for higher-risk patients, but short-term mortality after surgery is reported to be very low [22]. Similar to our study population, incidences were <1% in previous studies using machine-learning techniques to develop prediction models [8,9]. The performance of machine-learning models is sensitive to the distribution of the dataset. To overcome this, we validated our model by down-sampling the dataset so that the proportion of outcomes was balanced, and we showed that the model was still robust in the balanced dataset.

Another strength of this study was that it was the first to validate a developed machine-learning model with data from different centers, which enhances its generalizability. Although broad application remains challenging due to differences in electronic medical record systems between centers, our model relied on common risk factors that were widely evaluated during the preoperative period. Additionally, variables shown with strong effects were clinically explainable, which is essential for interpreting results from machine-learning techniques and is also advantageous for applying the model in daily practice. A model based on common risk factors can be adopted by centers without them needing to change their institutional protocols, and it is feasible for clinicians to rely on. Some blood laboratory test results included in the model are even modifiable, and improvement in these values can theoretically decrease risk. However, the potential to apply this model for improving clinical outcomes was not thoroughly investigated and requires further study.

Our study has several limitations. First, it used retrospective data, and causality cannot be confirmed regardless of clinical relevance. In addition, we retained patients regardless of surgery types, although surgery type itself largely affects mortality. Our prediction model for 30-day mortality may not be clinically meaningful for palliative procedures or life-critical emergency surgeries. Second, although the model was validated, it cannot be fully generalized, especially for other ethnicities. Additionally, perioperative care may not have been controlled. Institutional protocols may vary between departments and could have changed during the study period. Some clinical decisions were made at the discretion of the attending clinicians. Third, the dataset was imbalanced with a low incidence of the primary outcome. Lastly, our study cannot confirm whether factor modification could improve outcomes or if model adoption would be clinically helpful. Despite these limitations, our study demonstrated a preoperative prediction model for postoperative mortality, and we validated the model using a dataset from another center.

## 5. Conclusions

We developed a machine-learning model for predicting 30-day mortality after non-cardiac surgery based on preoperative variables automatically extracted from electronic medical records, and we validated the model in a multi-center setting. Implementation of the model may help clinicians predict postoperative outcomes.

## Figures and Tables

**Table 1:** Preoperative variables used in the prediction model.

Demographic Data	Sex
	Age
	Weight
	Height
Vital signs	Systolic blood pressure
	Diastolic blood pressure
	Respiratory rate
	Temperature
	Pulse rate
Underlying conditions	ASA physical status
	I
	II
	III
	IV
	V
	E
	Cerebrovascular disease
	Coronary artery disease
Physical examination	Respiratory difficulty
	Chest pain
	Cough
	Wheezing
	Pregnancy
Preoperative blood laboratory tests and electrolytes	Albumin
	Alanine aminotransferases
	Aspartate aminotransferases
	Activated partial thromboplastin time
	Blood urea nitrogen
	Creatinine
	Glucose
	Hemoglobin
	Hematocrit
	International Normalized Ratio
	Platelet
	Potassium
	Sodium

**Table 2:** Baseline characteristics of patients in the development dataset.

	Missing	Overall	0	1	*p*-Value
***n***		276,341	275,900	441	
**Male, *n* (%)**		120,296 (43.5)	120,011 (43.5)	285 (64.6)	<0.001
**ASA Class 1, *n* (%)**		103,239 (37.4)	103,237 (37.4)	2 (0.5)	<0.001
**ASA Class 2, *n* (%)**		138,063 (50.0)	138,018 (50.0)	45 (10.2)	<0.001
**ASA Class 3, *n* (%)**		24,558 (8.9)	24,486 (8.9)	72 (16.3)	<0.001
**ASA Class 4, *n* (%)**		3245 (1.2)	3183 (1.2)	62 (14.1)	<0.001
**ASA Class 5, *n* (%)**		86 (0.0)	73 (0.0)	13 (2.9)	<0.001
**ASA Class 6, *n* (%)**		56 (0.0)	14 (0.0)	42 (9.5)	<0.001
**ASA Class E, *n* (%)**		7078 (2.6)	6874 (2.5)	204 (46.3)	<0.001
**Cerebrovascular disease, *n* (%)**		916 (0.3)	916 (0.3)		0.412
**Coronary artery disease, *n* (%)**		789 (0.3)	789 (0.3)		0.642
**Pregnant, *n* (%)**		2921 (1.1)	2869 (1.0)	52 (11.8)	0.082
**Respiratory difficulty, *n* (%)**		1936 (0.7)	1936 (0.7)		<0.001
**Chest pain, *n* (%)**		1313 (0.5)	1305 (0.5)	8 (1.8)	0.001
**Cough, *n* (%)**		548 (0.2)	546 (0.2)	2 (0.5)	0.218
**Wheezing, *n* (%)**		96 (0.0)	96 (0.0)		1
**Age, mean (SD)**	0	53.4 (15.3)	53.4 (15.3)	60.0 (16.0)	<0.001
**Weight, mean (SD)**	4481	64.6 (23.7)	64.6 (23.7)	64.4 (16.3)	0.885
**Height, mean (SD)**	5652	162.0 (15.1)	162.0 (15.1)	162.2 (14.0)	0.876
**Systolic blood pressure, mean (SD)**	0	120.5 (79.9)	120.5 (79.9)	123.6 (27.5)	0.087
**Diastolic blood pressure, mean (SD)**	0	71.1 (11.9)	71.1 (11.9)	71.5 (17.8)	0.696
**Pulse rate, mean (SD)**	0	71.7 (22.9)	71.6 (22.9)	92.6 (23.7)	<0.001
**Respiratory rate, mean (SD)**	0	18.5 (2.1)	18.5 (2.1)	19.2 (4.4)	0.017
**Temperature, mean (SD)**	0	36.4 (7.6)	36.4 (7.6)	36.5 (0.8)	0.213
**Albumin, mean (SD)**	2850	4.4 (0.5)	4.4 (0.5)	3.4 (0.7)	<0.001
**Alanine aminotransferase, mean (SD)**	1115	22.9 (51.5)	22.9 (51.3)	65.9 (165.2)	<0.001
**Activated partial thromboplastin time, mean (SD)**	1432	36.0 (8.9)	36.0 (8.9)	47.2 (24.2)	<0.001
**Aspartate aminotransferase, mean (SD)**	1109	28.1 (788.4)	28.0 (788.7)	102.5 (260.7)	<0.001
**Blood urea nitrogen, mean (SD)**	1184	14.9 (7.9)	14.9 (7.9)	25.7 (19.8)	<0.001
**Creatinine, mean (SD)**	1241	0.9 (1.0)	0.9 (1.0)	1.5 (1.5)	<0.001
**Glucose, mean (SD)**	4851	110.0 (34.4)	110.0 (34.4)	152.1 (70.9)	<0.001
**Hemoglobin, mean (SD)**	696	13.2 (1.9)	13.2 (1.9)	10.8 (2.5)	<0.001
**Hematocrit, mean (SD)**	736	39.7 (5.1)	39.7 (5.1)	32.4 (7.4)	<0.001
**International normalized ratio, mean (SD)**	1187	1.0 (0.3)	1.0 (0.3)	1.5 (0.8)	<0.001
**Platelet, mean (SD)**	742	242.0 (72.2)	242.0 (72.1)	160.6 (104.0)	<0.001
**Potassium, mean (SD)**	1335	4.2 (0.4)	4.2 (0.4)	4.1 (0.6)	0.008
**Sodium, mean (SD)**	1327	140.4 (2.6)	140.4 (2.6)	138.5 (7.5)	<0.001

Data are presented as *n* (%) or mean (±standard deviation). ASA: American Society of Anesthesiologists.

**Table 3:** Subgroup analysis by age and sex.

	Internal Validation Dataset			External Validation Dataset		
	AUROC	AUPRC	Patients (%)	AUROC	AUPRC	Patients (%)
**Age (years)**						
≥29	0.943 (0.896–0.999)	0.198 (0.001–0.657)	4324 (0.09)	0.954 (0.916–0.992)	0.194 (0.072–0.316)	17,681 (0.18)
30–39	0.977 (0.965–0.989)	0.295 (0.07–0.52)	6947 (0.12)	0.98 (0.964–0.996)	0.19 (0.039–0.341)	9355 (0.19)
40–49	0.97 (0.958–0.999)	0.401 (0.165–0.637)	10,286 (0.13)	0.942 (0.907–0.977)	0.17 (0.054–0.286)	12,153 (0.25)
50–59	0.959 (0.888–0.999)	0.394 (0.170–0.618)	20,159 (0.17)	0.943 (0.920–0.966)	0.186 (0.087–0.285)	11,188 (0.46)
≥60	0.91 (0.887–0.934)	0.106 (0.03–0.179)	13,000 (0.14)	0.849 (0.810–0.899)	0.071 (0.04–0.101)	14,187 (0.74)
**Sex**						
Male	0.94 (0.906–0.976)	0.228 (0.110–0.346)	23,798 (0.18)	0.931 (0.916–0.946)	0.132 (0.086–0.178)	32,311 (0.48)
Female	0.966 (0.953–0.979)	0.238 (0.106–0.36)	30,918 (0.11)	0.928 (0.904–0.951)	0.114 (0.052–0.176)	32,253 (0.25)

AUROC denotes the area under the receiver operating characteristic curve, and AUPRC indicates the area under the precision and recall curve. The numbers in parentheses mean 95% confidence intervals of AUROC and AUPRC obtained by 1000 times bootstrapping. “Patients” represents the number of patients, and the % next to “Patients” means the outcome’s occurrence rate.

## Data Availability

The data presented in this study are available on request from the corresponding author. The data are not publicly available due to institutional restrictions.

## Supplementary Materials

The following supporting information can be downloaded at: https://www.mdpi.com/article/10.3390/jcm11216487/s1, Table S1: Baseline characteristics of external validation dataset (Ajou University Medical Center). Table S2: Performance Metrics of Models. Figure S1: Performance of model in the balanced dataset.
