# Supplementary material for: Prediction Model for 30-Day Mortality after Non-Cardiac Surgery Using Machine-Learning Techniques Based on Preoperative Evaluation of Electronic Medical Records
Source: J Clin Med. 2022 Nov 1;11(21):6487. doi: 10.3390/jcm11216487 (PMC9659244; doi:10.3390/jcm11216487)
Supplement: Supplementary file 1 [file jcm-11-06487-s001.zip › suppletable1.pdf]

Supplementary Table S1. Baseline characteristics of external validation dataset (Ajou University Medical Center)

	Missing	Overall	No death	Death	P value
n		63384	63169	215	
Male, n (%)		31777 (50.1)	31638 (50.1)	139 (64.7)	<0.001
ASA Class 1, n (%)		41642 (65.7)	41624 (65.9)	18 (8.4)	<0.001
ASA Class 2, n (%)		16983 (26.8)	16933 (26.8)	50 (23.3)	0.273
ASA Class 3, n (%)		2198 (3.5)	2142 (3.4)	56 (26.0)	<0.001
ASA Class 4, n (%)		45 (0.1)	35 (0.1)	10 (4.7)	<0.001
ASA Class 5, n (%)		13 (0.0)	11 (0.0)	2 (0.9)	0.001
ASA Class 6, n (%)		6 (0.0)	1 (0.0)	5 (2.3)	<0.001
ASA Class E, n (%)		2300 (3.6)	2226 (3.5)	74 (34.4)	<0.001
Cerebrovasuclar disease, n (%)		2456 (3.9)	2445 (3.9)	11 (5.1)	0.443
Coronary artery disease, n (%)		2603 (4.1)	2590 (4.1)	13 (6.0)	0.206
Pregant, n (%)		1347 (2.1)	1347 (2.1)		0.065
Respiratory difficulty, n (%)		122 (0.2)	120 (0.2)	2 (0.9)	0.016
Chest pain, n (%)		197 (0.3)	195 (0.3)	2 (0.9)	0.144
Cough, n (%)		99 (0.2)	99 (0.2)		1
Wheezing, n (%)		0 (0.0)	0 (0.0)		1
Age, mean (SD)	0	41.8 (21.7)	41.8 (21.7)	55.6 (19.9)	<0.001
Weight, mean (SD)	3714	58.6 (23.2)	58.6 (23.3)	60.6 (16.0)	0.158
Height, mean (SD)	5622	156.3 (24.0)	156.3 (24.1)	162.1 (15.6)	<0.001
Systolic blood pressure, mean (SD)	0	123.9 (20.3)	124.0 (20.2)	114.8 (39.2)	0.001
Diastolic blood pressure, mean (SD)	0	77.2 (27.8)	77.2 (27.8)	65.4 (23.0)	<0.001
Pulse rate, mean (SD)	0	81.9 (28.4)	81.9 (28.4)	95.6 (32.3)	<0.001
Respiratory rate, mean (SD)	0	20.6 (15.2)	20.6 (15.1)	22.0 (25.4)	0.432
Temperature, mean (SD)	0	36.7 (1.7)	36.7 (1.6)	35.4 (5.5)	<0.001
Albumin, mean (SD)	2833	3.8 (0.6)	3.8 (0.6)	3.0 (0.9)	<0.001
Alanine aminotrasnferase, mean (SD)	3041	35.5 (71.7)	34.7 (58.1)	166.8 (530.2)	0.001
Activated partial thromboplastin time, mean (SD)	6256	36.4 (14.9)	35.6 (12.6)	61.6 (38.4)	0.002
Asparate aminotrasnferase, mean (SD)	3041	45.9 (130.2)	44.3 (78.2)	310.3 (1322.6)	0.005
Blood urea nitrogen, mean (SD)	3049	12.9 (7.2)	12.9 (7.1)	19.6 (13.7)	<0.001
Creatinine, mean (SD)	4983	0.9 (0.8)	0.9 (0.8)	1.3 (1.4)	<0.001
Glucose, mean (SD)	3140	129.7 (45.6)	129.3 (45.0)	191.0 (90.5)	<0.001
Hemoglobin, mean (SD)	4034	12.2 (1.9)	12.2 (1.9)	10.6 (2.8)	<0.001
Hematocrit, mean (SD)	2473	36.1 (5.6)	36.1 (5.6)	32.0 (8.3)	<0.001
International normalized ratio, mean (SD)	4573	1.1 (0.2)	1.1 (0.2)	1.5 (0.7)	<0.001
Platelet, mean (SD)	2645	217.3 (79.5)	217.7 (79.4)	156.9 (90.3)	<0.001
Potassium, mean (SD)	3077	4.0 (0.5)	4.0 (0.5)	4.0 (0.9)	0.857
Sodium, mean (SD)	3077	139.5 (2.9)	139.5 (2.9)	140.8 (6.3)	0.003
