# Supplementary material for: Prediction Model for 30-Day Mortality after Non-Cardiac Surgery Using Machine-Learning Techniques Based on Preoperative Evaluation of Electronic Medical Records
Source: J Clin Med. 2022 Nov 1;11(21):6487. doi: 10.3390/jcm11216487 (PMC9659244; doi:10.3390/jcm11216487)
Supplement: Supplementary file 1 [file jcm-11-06487-s001.zip › suppletable2.pdf]

Supplementary Table S2. Performance Metrics of Models

	AUROC	AUPRC
Extreme gradient boosting	0.960	0.216
Logistic Regression	0.851	0.070
Random Forest	0.888	0.149
Naive Bayes	0.867	0.105

AUROC: area under the receiver operating characteristic curve, AUPRC: area under the precision and recall curve.
