# Supplementary figures and images for: Prediction Model for 30-Day Mortality after Non-Cardiac Surgery Using Machine-Learning Techniques Based on Preoperative Evaluation of Electronic Medical Records
Source: J Clin Med. 2022 Nov 1;11(21):6487. doi: 10.3390/jcm11216487 (PMC9659244; doi:10.3390/jcm11216487)

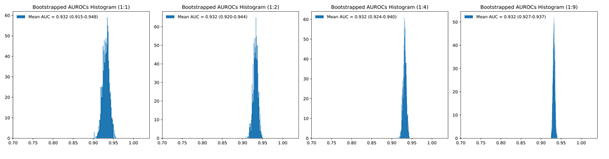

Supplement: Supplementary file 1 [file jcm-11-06487-s001.zip › supplefig1.tif]
